# Genetics of Tinnitus: Still in its Infancy

**DOI:** 10.3389/fnins.2017.00236

**Published:** 2017-05-08

**Authors:** Barbara Vona, Indrajit Nanda, Wafaa Shehata-Dieler, Thomas Haaf

**Affiliations:** ^1^Institute of Human Genetics, Julius Maximilians University WürzburgWürzburg, Germany; ^2^Plastic, Aesthetic and Reconstructive Surgery, Department of Otorhinolaryngology, Comprehensive Hearing Center, University Hospital WürzburgWürzburg, Germany

**Keywords:** complex disorders, genetics, genetic heterogeneity, genome-wide association study (GWAS), hearing loss, tinnitus, twin study

## Abstract

Tinnitus is the perception of a phantom sound that affects between 10 and 15% of the general population. Despite this considerable prevalence, treatments for tinnitus are presently lacking. Tinnitus exhibits a diverse array of recognized risk factors and extreme clinical heterogeneity. Furthermore, it can involve an unknown number of auditory and non-auditory networks and molecular pathways. This complex combination has hampered advancements in the field. The identification of specific genetic factors has been at the forefront of several research investigations in the past decade. Nine studies have examined genes in a case-control association approach. Recently, a genome-wide association study has highlighted several potentially significant pathways that are implicated in tinnitus. Two twin studies have calculated a moderate heritability for tinnitus and disclosed a greater concordance rate in monozygotic twins compared to dizygotic twins. Despite the more recent data alluding to genetic factors in tinnitus, a strong association with any specific genetic locus is lacking and a genetic study with sufficient statistical power has yet to be designed. Future research endeavors must overcome the many inherent limitations in previous study designs. This review summarizes the previously embarked upon tinnitus genetic investigations and summarizes the hurdles that have been encountered. The identification of candidate genes responsible for tinnitus may afford gene based diagnostic approaches, effective therapy development, and personalized therapeutic intervention.

## Introduction

Tinnitus is described as a scientific and clinical enigma that affects 10–15% of the general population. Furthermore, ~1–3% of the population can be diagnosed with debilitating tinnitus connected to sleep disturbances, psychiatric distress, and quality of life consequences (Deniz et al., 2010; Shargorodsky et al., 2010; Baguley et al., 2013). Without question, the personal and societal strain from debilitating tinnitus can be enormous. The American Tinnitus Society describes the annual personal financial burden of tinnitus to be as high as $30,000 from compounded healthcare costs, lost income, and reduced productivity (https://www.ata.org/understanding-facts/impact-tinnitus).

Tinnitus is perceived as ringing, buzzing, beeping, or hissing and is characterized according to various clinical criteria. It can be subjective (perceived by the affected individual) or objective (heard by an observer), continuous or episodic, unilateral or bilateral, or pulsatile (synchronous or asynchronous). It can range from low- to high-intensity sound and can manifest any frequency. Tinnitus can be acute (<3 months), sub-acute (3–6 months), or chronic (>12 months) with a gradual or sudden onset or be associated with other triggers or comorbidities (Baguley et al., 2013). In combination, these features complicate precise tinnitus phenotyping and have hampered research aiming to uncover a genetic basis for tinnitus.

Risk factors of tinnitus include hearing loss, sound exposure, stress, anxiety, depression, ototoxic drugs, hypertension, and aging. While the association between individual risk factors and tinnitus is not straightforward, tinnitus seems to be correlated with advancing age and hearing loss (Baguley et al., 2013). Interestingly, only about half of patients with tinnitus have recognized risk factors, which is a reason it has been hypothesized that predisposition to tinnitus is linked with genetic background (Shargorodsky et al., 2010).

Secondary tinnitus has been conventionally recognized as a symptom of a variety of monogenic disorders for which many genes or loci have already been identified (Table 1). In contrast, recognition of chronic primary tinnitus may be obscured by non-Mendelian inheritance patterns, which contribute to a lack of awareness and underreporting of tinnitus within families and among relatives (Sand et al., 2007). The association between genetic factors and primary tinnitus has historically lacked consensus and replication. Tinnitus could result from a number of pathological processes involving peripheral (cochlear) and/or central auditory abnormalities. The lack of consensus concerning these mechanisms asserts that further research is required.

**Table 1:** **Monogenic disorders associated with secondary tinnitus with variable onset and severity**.

**Gene**	**DFN Locus**	**MIM**	**Gene function**	**Disorder**	**References**
*ACTG1*	DFNA20/26	102560	Actin gamma 1	Autosomal dominant non-syndromic hearing loss	de Heer et al., 2009
*AIFM1*	AUNX1	300169	Apoptosis inducing factor, mitochondria associated 1	X-linked non-syndromic hearing loss	Wang et al., 2006; Zong et al., 2015
*ANKH*	−	605145	ANKH inorganic pyrophosphate transport regulator	Craniometaphyseal dysplasia	Kornak et al., 2010
*ATP1A2*	−	182340	ATPase Na^+^/K^+^ transporting subunit alpha 2	Familiar basilar migraine	Ambrosini et al., 2005
*CACNA1A*	−	601011	Calcium channel, voltage-dependent, P/Q type, alpha-1A subunit	Episodic ataxia type II	Wan et al., 2011
*CEACAM16*	DFNA4B	614591	Carcinoembryonic antigen related cell adhesion molecule 16	Autosomal dominant non-syndromic hearing loss	Wang et al., 2015
*COCH*	DFNA9	603196	Cochlin	Autosomal dominant non-syndromic hearing loss	Gallant et al., 2013
*COL1A1*	−	120150	Collagen, type I, alpha-1	Osteogenesis imperfecta type I	Kuurila et al., 2003
*COL1A2*	−	120160	Collagen, type I, alpha-2	Osteogenesis imperfecta	Kuurila et al., 2003
*DIABLO*	DFNA64	605219	Diablo IAP-binding mitochondrial protein	Autosomal dominant non-syndromic hearing loss	Cheng et al., 2011
*DSPP*	−	125485	Dentin sialophosphoprotein	Dentinogenesis imperfecta with or without progressive hearing loss	Xiao et al., 2001
*DTNA*	−	601239	Dystrobrevin alpha	Autosomal dominant familial Ménière disease	Requena et al., 2015
*FAM136A*	−	616275	Family with sequence similarity 136 member A	Autosomal dominant familial Ménière disease	Requena et al., 2015
*GJB2*	DFNA3A	121011	Gap junction protein beta-2	Autosomal dominant non-syndromic hearing loss	Wang et al., 2017
*GJB2*	DFNB1	121011	Gap junction protein beta-2	Autosomal recessive non-syndromic hearing loss	Dodson et al., 2011
*GJB3*	DFNA2A	603324	Gap junction protein beta-3	Autosomal dominant non-syndromic hearing loss	Coucke et al., 1994; Xia et al., 1998
*GLA*	−	300644	Galactosidase, alpha	Fabry disease	Germain et al., 2002; Conti and Sergi, 2003
*JAK2*	−	147796	Janus kinase 2	Polycythemia vera	Mihalj et al., 2013
*KCNQ4*	DFNA2A	603537	Potassium channel, voltage-gated channel subfamily member 4	Autosomal dominant non-syndromic hearing loss	Kubisch et al., 1999
*MFN2*	−	608507	Mitofusin 2	Hereditary motor and sensory neuropathy VI	Voo et al., 2003
*MIR96*	DFNA50	611606	MicroRNA 96	Autosomal dominant non-syndromic hearing loss	Modamio-Høybjør et al., 2004; Mencía et al., 2009
*MT-TS1*	−	590080	Mitochondrially encoded tRNA serine 1	Mitochondrial non-syndromic hearing loss	Chapiro et al., 2002
*MT-RNR1*	−	561000	Mitochondrially encoded 12S RNA	Mitochondrial non-syndromic hearing loss	Matsunaga et al., 2004; Bravo et al., 2006
*MYO7A*	DFNA11	276903	Myosin VIIA	Autosomal dominant non-syndromic hearing loss	Sun et al., 2011
*NAGA*	−	104170	Alpha-N-acetylgalactosamineidase	Kanzaki disease	Kodama et al., 2001
*NF2*	−	607379	Neurofibromin 2	Neurofibromatosis type 2	Evans et al., 1992
*OSBPL2*	DFNA67	606731	Oxysterol-binding protein-like protein 2	Autosomal dominant non-syndromic hearing loss	Xing et al., 2015
*P2RX2*	DFNA41	600844	Purinergic receptor P2X 2	Autosomal dominant non-syndromic hearing loss	Yan et al., 2013
*PRKCB*	−	176970	Protein kinase C beta	Autosomal dominant familial Ménière disease	Martín-Sierra et al., 2016
*PRPS1*	DFNX1	311850	Phosphoribosyl pyrophosphate synthetase 1	X-linked non-syndromic hearing loss	Liu et al., 2010
SDHB^*^	−	185470	Succinate dehydrogenase complex, subunit B, iron sulfur protein	Paragangliomas 4	Bayley et al., 2006; Sagong et al., 2016
SDHC^*^	−	602413	Succinate dehydrogenase complex, subunit C, integral membrane protein, 15-KD	Paragangliomas 3	Bickmann et al., 2014
SDHD^*^	−	602690	Succinate dehydrogenase complex, subunit D, integral membrane protein	Paragangliomas 1	Badenhop et al., 2001; Tan et al., 2009
*TMC1*	DFNA36	606706	Transmembrane cochlear expressed gene 1	Autosomal dominant non-syndromic hearing loss	Zhao et al., 2014
*VHL*	−	608537	von Hippel-Lindau tumor suppressor	von Hippel-Lindau syndrome	Butman et al., 2007
*WFS1*	DFNA6/14/38	606201	Wolframin ER transmembrame glycoprotein	Autosomal dominant non-syndromic hearing loss, low-frequency hearing loss	Lesperance et al., 1995
Unknown	DFNA16	603964	−	Autosomal dominant non-syndromic hearing loss	Fukushima et al., 1999
Unknown	DFNA33	614211	−	Autosomal dominant non-syndromic hearing loss	Bönsch et al., 2009
Unknown	DFNA43	608394	−	Autosomal dominant non-syndromic hearing loss	Flex et al., 2003
Unknown	DFNA57	−	−	Autosomal dominant non-syndromic hearing loss	Bönsch et al., 2008
Unknown	DFNA58	615654	−	Autosomal dominant non-syndromic hearing loss	Lezirovitz et al., 2009
Unknown	DFNY1	400043	−	Y-linked hearing loss	Wang et al., 2013

* *Pulsatile tinnitus (tympanic paraganglioma) associated*.

Identification of genetic factors would provide important insights into the pathogenesis of tinnitus, facilitate understanding of the course and severity of tinnitus burden on patients, and permit novel diagnostic strategies. The majority of research investigations dissecting the genetics of tinnitus have taken the form of association studies that have revealed few borderline-significant results (Table 2). Recently, a genome-wide association study (GWAS) has identified potential metabolic pathways meriting further investigation (Gilles et al., 2017) and two twin-study cohorts have uncovered heritability estimates that provide pioneering insight into moderate genetic influences for tinnitus (Bogo et al., 2016; Maas et al., 2017). Although an excellent review discussing the genetics of tinnitus that touches upon phenotyping strategies and proposed pathophysiological mechanisms has been recently published (Lopez-Escamez et al., 2016), the present review exclusively emphasizes the genetic studies that have been published to date, discusses emerging data that suggests a complex or multifactorial genetic etiology, and presents an outlook for future research.

**Table 2:** **Genes screened in tinnitus candidate gene studies**.

**Gene**	**MIM**	**Gene function**	**Number of subjects**	**Phenotype**	**Method**	**Outcome**	**Stastical power**	**Multiple testing correction**	**References**
**CARDIOVASCULAR ASSOCIATED GENES**									
*ACE*	106180	Angiotensin I converting enzyme	89	Severe chronic tinnitus	PCR-RFLP genotyping	-No significance	Not described	Not described	Yüce et al., 2016
*ADD1*	102680	Adducin 1	89	Severe chronic tinnitus	PCR-RFLP genotyping	-The p.G460W heterozygous genotype (*p* = 0.009) and W allele (*p* = 0.021) are statistically significantly higher in patients than controls	Not described	Not described	Yüce et al., 2016
**NEUROTROPHIC FACTORS**									
*BDNF*	113505	Brain derived neurotrophic factor	240	Chronic tinnitus	PCR-RFLP genotyping	-No correlation between tinnitus and rs2049046 and rs6265 polymorphisms	Underpowered, nearly 15,000 patients and >680,000 controls required for exclusion of a modifying risk from rs6265	Yes	Sand et al., 2012b
*GDNF*	600837	Glial cell derived neurotrophic factor	52	Chronic tinnitus	PCR-RFLP genotyping	-No correlation between tinnitus and rs884344, rs3812047 and rs1110149 polymorphisms -Heterozygosity was significantly lower (*p* = 0.02) for rs1110149 between patients and controls	Not described	Not described	Orenay-Boyacioglu et al., 2016
			240	Chronic tinnitus	PCR-RFLP genotyping	-No correlation between tinnitus and rs1110149, rs884344 and rs3812047 polymorphisms	Underpowered	Yes	Sand et al., 2012b
**POTASSIUM RECYCLING PATHWAY GENES**									
*KCNE1*	176261	Potassium voltage-gated channel subfamily E regulatory subunit 1	201	Chronic tinnitus	Sanger sequencing	-No correlation between tinnitus severity and 46 polymorphic variants -V47I novel variant detected	Underpowered, >12,500 patients required to exclude a modifying allele risk	No	Sand et al., 2010
			128	Noise exposed males with tinnitus	SNP genotyping	-Significance was detected in rs915539 (*p* = 0.005) in noise-resistant subjects and when comparing tinnitus patients vs. controls in noise-resistant and susceptible groups (*p* = 0.018)	Not described	No	Pawełczyk et al., 2012
*KCNE3*	604433	Potassium voltage-gated channel subfamily E regulatory subunit 3	288	Chronic tinnitus	Sanger sequencing	-No association between tinnitus and 11 polymorphic variants	Underpowered, 2,707 patients and 65,083 controls required	No	Sand et al., 2011
*SLC12A2*	600840	Solute carrier family 12 member 2	128	Noise exposed males with tinnitus	SNP genotyping	-Significance was detected in rs10089 (*p* = 0.016) in noise susceptible subjects and when comparing tinnitus patients vs. controls in noise-resistant and susceptible groups (*p* = 0.026)	Not described	No	Pawełczyk et al., 2012
**GABA_B_ RECEPTOR SUBUNIT**									
*KCTD12*	610521	Potassium channel tetramerization domain containing 12	95	Chronic tinnitus	Sanger sequencing	-rs34544607 was associated with tinnitus (*p* = 0.04) but weakened after screening 50 additional cases (*p* = 0.07)	Underpowered, 363 tinnitus cases required	No	Sand et al., 2012a
						-Gene did not predict tinnitus severity			
**SEROTONIN RECEPTOR/TRANSPORTER**									
*HTR1A*	109760	5-hydroxytrypthamine receptor 1A	88	Chronic tinnitus	Sanger sequencing	-No correlation between tinnitus and rs1800043 polymorphism	Not described	Not described	Kleinjung et al., 2006
*SLC6A4*	182138	Solute carrier family 6 member 4	54	Chronic tinnitus	PCR and VNTR analysis	-Association between quality of life scores (severity, *p* = 0.004; tinnitus discomfort level, *p* = 0.002; attention deficit, *p* = 0.04; sleep disorder, *p* = 0.04) and patients with the 5-HTTLPR polymorphism	Not described	Not described	Deniz et al., 2010

PCR-RFLP: polymerase chain reaction-restriction fragment length polymorphism; VNTR: variable number tandem repeat

*The study from Pawełczyk et al. also included the analysis of eight further genes involved in the potassium recycling pathway (GJB1, GJB2, GJB3, GJB4, GJB6, KCNJ10, KCNQ1, KCNQ4) but results were not significant (Pawełczyk et al., 2012)*.

## Complex genetics approaches

Heritability of tinnitus is defined as tinnitus variance explained by additive genetic factors. The earliest attempts of estimating the heritability of tinnitus stemmed from large family-based questionnaire studies. One of these studies assessed familial aggregation in seven European countries that proposed a sibling-sibling tinnitus correlation of 0.16 in 981 siblings and an increased 1.7-fold likelihood of developing tinnitus with an affected sibling. However, the authors rationalized that this could be due to increased tinnitus awareness within families (Hendrickx et al., 2007). Another study analyzed questionnaire data in Norwegian nuclear families and considered genetic and environmental effects in subjects reporting tinnitus (Kvestad et al., 2010). Heritability estimates returned an upper limit value of 0.11. Criticisms of this study remarked on a lack of attention to questionnaire design and phrasing. Additionally, although the study was conducted as a family-based approach, tinnitus sub-typing of the 28,066 participating individuals was not undertaken and similar replication studies have not followed (Sand, 2011).

**Figure 1:**
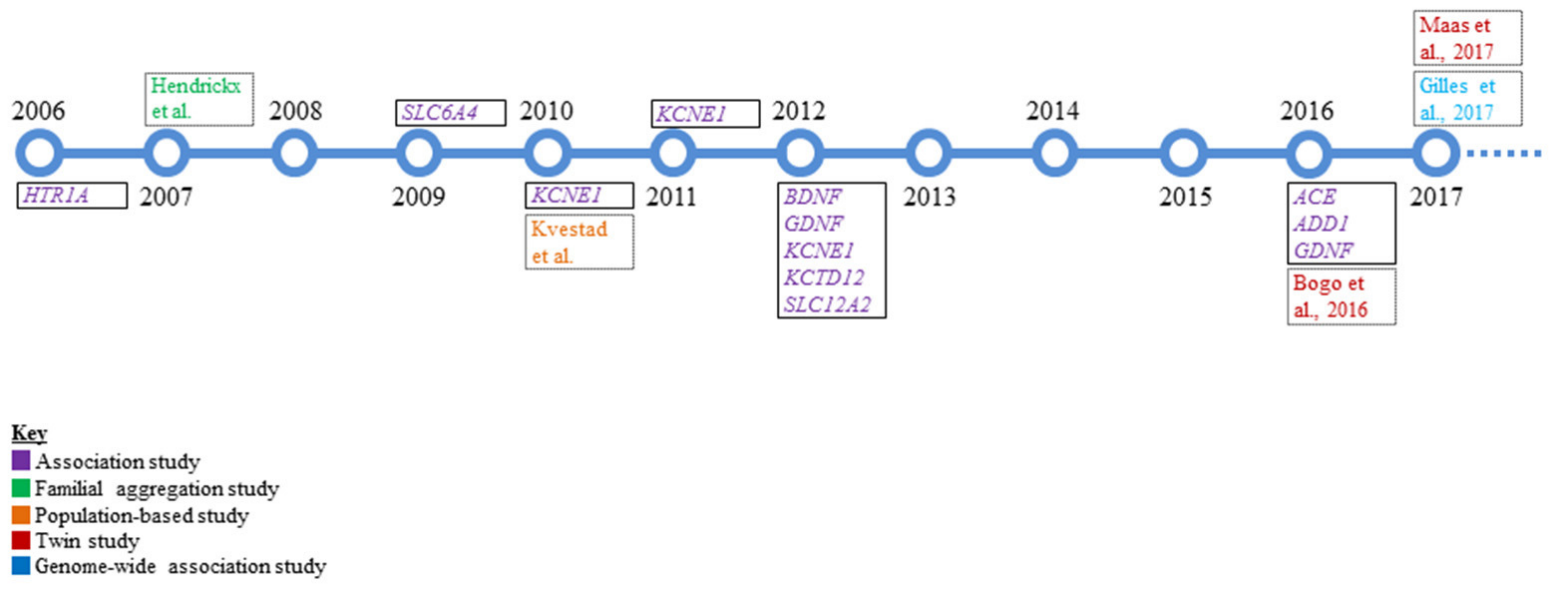
**A timeline overview of the genetic research in tinnitus that has been conducted to date**. Single genes that were studied via candidate gene association analysis are boxed with a solid black line and represent the majority of work performed in the genetics of tinnitus. All other studies are boxed with a dotted black line.

Despite the considerable prevalence of tinnitus, the lack of Mendelian inheritance and genetic factors implicated from these early studies support that tinnitus is a complex trait. The identification of complex disease alleles is very challenging in the presence of potential genotype-by-environment interaction, incomplete penetrance, environmental phenocopies, genetic heterogeneity, or polygenic inheritance (Lander and Schork, 1994; Silverman and Palmer, 2000). Complex genetic disorders result from relatively common variants in multiple genes that each contribute effects of varying magnitude and are connected to variants that predispose an individual to a disorder rather than directly causing it (Zondervan and Cardon, 2007). Genetic dissection of tinnitus has followed several different paths that include candidate gene association, twin, and GWAS that are summarized below and in Figure 1.

### Association studies

Association studies can take the form of hypothesis-driven candidate gene or hypothesis-free GWAS, with the latter described in a subsequent section in this review. Case-control association testing compares genotype frequencies between unaffected and affected individuals and takes considerable differences between these two groups as evidence for or against disease susceptibility. Association studies in complex disorders can yield useful information if findings are replicated, or alternatively, if associations are confirmed with linkage analyses studying large families. Examples of replicated association findings are the discovery of the genes *ANXA11* and *BTNL2* in sarcoidosis and *DTNBP1* and *NRG1* in schizophrenia (Riley and Kendler, 2006; Spagnolo and du Bois, 2007). However, such findings are rather rare and many studies run the risk of artefactual positive association due to case-control selection bias, population admixture, or alleles residing in linkage disequilibrium (LD) with an allele directly affecting phenotype expression. Furthermore, in candidate gene association studies, the actual gene(s) of interest must already be identified for sequencing or genotyping. Late-onset disorders make selecting control groups challenging and can present a problem in young asymptomatic or undiagnosed individuals with risk alleles (Silverman and Palmer, 2000).

Case-control association testing has been a relatively widely employed approach and has comprised the majority of genetics research conducted in tinnitus patients to date (Table 2, Figure 1). Tinnitus candidate gene selection has included genes enriched in cardiovascular function (*ACE, ADD1*), neurotrophic factors (*BDNF, GDNF*), ion recycling pathways (*KCNE1, KCNE3, SLC12A2*), GABA_B_ receptor subunit (*KCTD12*), and serotonin receptor/transporter (*HTR1A, SLC6A4*) function. Presently, nine case-control studies have examined a combined total of 18 genes that are summarized in Table 2 (Kleinjung et al., 2006; Deniz et al., 2010; Sand et al., 2010, 2011, 2012a,b; Pawełczyk et al., 2012; Orenay-Boyacioglu et al., 2016; Yüce et al., 2016). For brevity, we describe selected case-control association studies with potentially significant results.

### Cardiovascular-associated gene

*ADD1* encodes ubiquitously expressed alpha-adducin. A well-studied polymorphism (p.G460W) has been linked to cardiovascular disease and hypertension (Staessen and Bianchi, 2005). Hypertension-associated auditory primary lesion sites are the organ of Corti and stria vascularis (Gates et al., 1993). An association study investigated the relationship between severe chronic tinnitus and the p.G460W polymorphism in 89 patients with severe chronic tinnitus and 104 age-matched Turkish-Caucasian controls (Yüce et al., 2016). Clinical tinnitus evaluation and severity assessment were performed by the Structured Tinnitus Interview and the Tinnitus Handicap Inventory, respectively. PCR-based restriction fragment length polymorphism (RFLP) analysis of the *ADD1* GW genotype (*p* = 0.009, χ^2^ = 9.4) and the W allele (*p* = 0.021, χ^2^ = 5.3) revealed significantly increased allele frequencies in the patient group (Yüce et al., 2016). This study asserted the potential involvement of the p.G460W genotype and W allele in *ADD1* in tinnitus pathophysiology.

### Neurotrophic factors

Tinnitus is thought to stem from central nervous system hyperexcitability and auditory cortical neuronal plasticity. Accumulating evidence indicates that tinnitus adaptation is dependent on cortical tonotopic map remodeling (Eggermont, 2016). An understanding of neurotrophins as important drivers of neural circuit remodeling in the auditory pathway have rationalized their relevance as candidate genes for tinnitus (Tan et al., 2007; Sand et al., 2012b).

PCR-based RFLP analysis in 240 German patients with Tinnitus Questionnaire (TQ)-scored subjective chronic primary tinnitus (Goebel and Hiller, 1994) has been performed for the genes *BDNF* and *GDNF*, encoding brain and glial cell-derived neurotrophic factors, respectively (Sand et al., 2012b). Both genes are essential in early central auditory pathway development. Two and three markers were investigated in *BDNF* (rs2049046 and rs6265) and *GDNF* (rs1110149, rs884344, and rs3812047), respectively. Comparison with reference data did not show significance after multiple testing correction; however, the authors could not exclude a weak modulatory effect. Furthermore, questionnaire intensity scores did not correlate with genetic variants, although notably, age-corrected multiple regression models with joint *BDNF* and *GDNF* genotypes indicated tinnitus severity could be predicted in women (*p* = 0.04, uncorrected; Sand et al., 2012b).

These three *GDNF* markers were similarly screened in a replication study including 52 Turkish patients with chronic tinnitus and 42 controls aged between 18 and 55 years (Orenay-Boyacioglu et al., 2016). No statistically significant distribution was detected in allele frequencies for all three markers between tinnitus and control groups. The only parameter reaching significance was heterozygosity (C:G) in the SNP rs1110149 (*p* = 0.02, χ^2^), that was found to have a lower frequency in tinnitus patients compared to controls (Orenay-Boyacioglu et al., 2016).

### Potassium recycling pathway genes

Pharmacological research has highlighted ion regulation and transport as potential therapeutic targets (Sand et al., 2011). Voltage-gated ion channels that are involved in auditory neural transmission by regulating endocochlear potentials are intriguing for exploration of tinnitus pathophysiology (Sand et al., 2010). The genes *KCNE1* and *SLC12A2* each encode a homologous β-potassium channel subunit and an inner ear Na^+^/2Cl^−^/K^+^ co-transporter, respectively, which have been screened in case-control association studies (Sand et al., 2010; Pawełczyk et al., 2012).

*KCNE1* screening in 201 Caucasian chronic TQ-scored tinnitus patients detected four coding and three non-coding variants, including one novel p.Val47Ile substitution and another novel 3′ UTR variant that were concluded as having a non-significant (*p* = 0.05, Fisher's exact test) dominant genotype or compound genotype effect without correction for multiple testing (Sand et al., 2010). These variants were not found to be causal by themselves or in compound heterozygosity for chronic TQ-scored tinnitus.

In a Polish genotyping study that included 10 potassium recycling genes, 128 noise-exposed subjects with tinnitus, and 498 noise-exposed controls responded to a questionnaire and underwent analysis. Case and control groups were divided into noise-resistant (normal audiograms) and noise-susceptible (abnormal audiograms) groups and individuals with a family history of hearing loss and other clinical indications or medication exposures were excluded. *KCNE1* and *SLC12A2* were associated with tinnitus based on significance in only one genetic marker per gene (rs915539 in *KCNE1, p* = 0.018; rs10089 in *SLC12A2, p* = 0.026). *p*-values were not subjected to multiple testing correction and, therefore, suggested as nominally significant results (Pawełczyk et al., 2012).

### GABA_B_ receptor subunit

Abundant data support chronic tinnitus with neuronal hyperactivity at different levels of the central auditory pathway, making drugs that increase inhibitory neurotransmission or block excitatory neurotransmission candidates for the treatment of tinnitus, and genes encoding these respective receptor complexes of potential interest (Eggermont and Roberts, 2004; Wang et al., 2011; Smith et al., 2012; Sand et al., 2012a). The gene *KCTD12* encodes a potassium channel tetramerization domain-containing protein that is tightly associated with the GABA_B2_ receptor carboxy-terminus (Sand et al., 2012a) and was subsequently subjected to association testing. The genomic DNAs from 95 German chronic TQ-scored tinnitus patients were obtained and the *KCTD12* open reading frame and adjacent 3′ untranslated regions were sequenced. Two rare synonymous and non-coding heterozygous variants were detected. Further, analysis disclosed one significant tinnitus-associated variant (rs34544607; *p* = 0.04, Fisher's exact test), but this significance weakened after screening 50 additional cases (*p* = 0.07, Fisher's exact test). No novel variants were detected and no variants were correlated with or predicted intensity of tinnitus; however, the authors acknowledge the study was underpowered.

### Serotonin transporter

There is considerable overlap in patients reporting disabling tinnitus in conjunction with other comorbidities and a particularly strong association among patients with comorbid depressive disorder that affects ~5–10% of the general population. There is an estimated 30% concordant overlap between comorbid depressive disorder and tinnitus that implies common molecular mechanisms and, therefore, overlapping genes attributing to both phenotypes (Tyler et al., 2006). As such, genes involved in serotonin regulation, a critical process associated with depressive psychiatric disorders, have been proposed as tinnitus candidate genes. Serotonin is present in hair cells, eighth nerve fibers, brainstem auditory nuclei and nuclei of the lateral lemniscus and superior olivary complex (Tyler et al., 2006).

The gene *SLC6A4* regulates serotonin neurotransmission and has been evaluated for tinnitus-association (Tyler et al., 2006; Deniz et al., 2010). A functional 5′-HTTLPR 44 base pair insertion-deletion polymorphism in the promoter region has been implicated in major depressive disorder (Hoefgen et al., 2005). This polymorphism and a 17 base pair variable number tandem repeat region in intron 2 were screened in 54 patients with subjective tinnitus and 174 population-matched controls. Tinnitus severity and psychoacoustic characteristics were assessed using the Beck Depression Inventory and visual analog scale, respectively. A significant association was detected between the 5′-HTTLPR polymorphism and visual analog scores that measured tinnitus quality of life impact using χ^2^ tests (severity, *p* = 0.004; tinnitus discomfort level, *p* = 0.002; attention deficit, *p* = 0.04; sleep disorder, *p* = 0.04). This study linked a polymorphism in the *SLC6A4* promoter with neurophysiological symptoms in tinnitus patients (Deniz et al., 2010).

### GWAS

GWAS can be powerful for the association of common variants and genetic loci in complex disorders and are appropriate for dissection of the “common disease-common variant” hypothesis. This hypothesis assumes a significant proportion of phenotypic divergence arises from common variants, typically with a minor allele frequency >5%, and that these variants are important for disease susceptibility (Sharma et al., 2014).

GWAS utilizes up to several million SNP genotypes most commonly generated from genotyping arrays to tag haplotype blocks, occasionally spanning more than 100 kb, on which functional variants reside. These studies utilize non-random co-inheritance of variants in linkage disequilibrium (LD) to test for case-control trait association (Edwards et al., 2013). *p*-value thresholds for statistical significance are very rigorous, typically below 10^−9^, to reduce the likelihood of false positive results, accommodate multiple testing burden, and provide enough stringency in studies that include lower frequency variants (minor allele frequency >5%; LaFramboise, 2009; Fadista et al., 2016). The conclusion after a successful GWAS is that one or more tag SNPs co-reside on haplotype blocks with variants having a biological function related to the phenotype. Interestingly, over 90% of disease-associated variants from GWAS reside in non-coding regions associated with transcriptional regulatory mechanisms involving promoter and enhancer element modulation (Maurano et al., 2012; Edwards et al., 2013). Following detection of statistically significant association signals, replication and functional experiments are required. A deeper understanding of these variants in a biological context requires experiments analyzing pathogenicity mechanisms such as transcriptional regulation, non-coding RNA function, and epigenetic regulation (Edwards et al., 2013). Functional assessment includes expression quantitative trait loci testing, *in vitro* protein and chromatin-structure assay analysis, as well as model organism experiments (Lee et al., 2014).

The first cross-sectional pilot tinnitus GWAS in ethnically homogeneous individuals between 55 and 65 years old was performed using 167 individuals with tinnitus and 749 non-tinnitus controls from Belgium (Gilles et al., 2017). These patients were previously included in a GWAS for age-related hearing loss in which a polygenic architecture was detected (Fransen et al., 2015). The association between tinnitus phenotype and 4,000,000 SNPs was tested and a gene-set enrichment analysis followed. 3.2% of the phenotypic variance was due to additive genetic effects. Although none of the SNPs reached genome-wide significance, potentially attributed to the limited sample size, the most interesting associations were revealed in the gene-set enrichment analysis that showed significance in seven metabolic pathways. The three most prominent pathways detected were the nuclear factor erythroid 2 like 2 (*NRF2*)-mediated oxidative stress response, endoplasmic reticulum (ER) stress response and serotonin reception mediated signaling pathways with low FDR-corrected *p*-values ranging from 0.004 to 0.02. NRF2-mediated oxidative stress plays a role in noise-induced hearing loss and tinnitus. Interestingly, tinnitus patients have been identified with substantially increased oxidative index levels compared to controls (Delmaghani et al., 2015; Koç et al., 2016). Moreover, ER stress has been associated with hearing loss via apoptosis (Van Rossom et al., 2015; Xue et al., 2016). Delayed hearing loss progression has been shown in transgenic mice with ER stress inhibitor treatment (Hu et al., 2016). Evidence of serotonin receptor mediated pathway involvement has been proposed from an apparent beneficial outcome of antidepressant usage among tinnitus patients (Baldo et al., 2012). Other pathways reaching significance include RAS, vascular smooth muscle contraction, coenzyme A biosynthesis, and NDK dynamin pathways (Gilles et al., 2017). Several limitations described by the authors included limited power to detect significant individual tinnitus-associated SNPs, the sample set was not selectively enriched for tinnitus patients and comprehensive controlling for risk factors was not undertaken. However, insight into seven potentially implicated pathways in tinnitus was highlighted from gene-set enrichment analysis.

### Twin studies

Twin-based epidemiological studies serve as a means to estimate heritability by comparing disease concordance in monozygotic (MZ) vs. dizygotic (DZ) twins. Increased concordance in genetically identical MZ vs. DZ twins, who share on average half of their alleles, suggests a role for genetic factors. It is assumed both MZ and DZ twins share the same family environment, thus yielding important information about the contribution of genetic factors to disease etiology. Two recent twin studies have been published. While there are differences in the experimental approaches that are detailed below, they independently concluded that genetic factors contribute to tinnitus.

A twin study by Bogo and colleagues evaluated the genetic effects of self-reported tinnitus in male twins aged 52–96 years who were included in a previous longitudinal study (Bogo et al., 2015) that analyzed genetic influence of age-related hearing loss. Male MZ and DZ twins who were born between 1914 and 1958 were included in baseline (*n* = 1084 individuals) and follow-up (*n* = 576 individuals) assessments 18 years apart that included audiometry and self-reported answers to questions about tinnitus status and severity. The hypothesis was that individuals with faster hearing deterioration had the greatest tinnitus risk and that genetic factors influenced tinnitus (Bogo et al., 2016).

No difference in tinnitus prevalence between MZ and DZ twins at either time point was detected and those who reported tinnitus disclosed a mild severity. Individuals (*n* = 576) were placed in one of four categories (never reported tinnitus, *n* = 361; tinnitus only at baseline, *n* = 24; tinnitus only at follow-up, *n* = 139; and tinnitus at both time points, *n* = 52). Those who reported tinnitus at baseline and both baseline and follow-up assessments showed remarkably poorer hearing at follow-up across all frequencies compared to the reference group (those never reporting tinnitus). Those with tinnitus only at follow-up did not have significantly different hearing thresholds compared to the reference group. MZ twin concordant rates were much higher than for DZ twins at both time points (baseline: MZ 0.46; DZ 0.07; follow-up: MZ 0.51; DZ 0.32), proposing that genetic factors were important. A genetic correlation measuring the extent of genetic influences correlated in tinnitus and hearing thresholds ranged from 0.33 to 0.49, and suggested a partial overlap of genes associated with tinnitus and hearing loss; however, the authors also concluded that most of the genetic variation in tinnitus was unique to tinnitus and not associated with co-occurring hearing loss. There was a greater hearing threshold difference between discordant DZ twin-pairs compared to MZ twin-pairs in cases and controls. Interestingly, the hearing thresholds among MZ twins discordant for tinnitus were more similar than for discordant DZ twins, which may be due to genetic background. Compared to controls, individuals with tinnitus have statistically significant hearing threshold shifts. An overall heritability of 0.4 was calculated, which demonstrates a moderate genetic influence on tinnitus. The authors disclosed that their study was underpowered, and that noise exposure and other risk factors were not assessed (Bogo et al., 2016).

Another twin study by Maas and colleagues took a slightly different approach and controlled for tinnitus laterality but did not assess hearing thresholds (Maas et al., 2017). Cross-sectional data from the Swedish Twin Registry, that includes participants from the “Screening Across the Lifespan Twin study” and the “Study of Twin Adults: Genes and Environment” (Magnusson et al., 2013) who were born between 1900 and 1985. Concordance rates between MZ and DZ twin-pairs (*n* = 10,464 twin pairs) were assessed. As opposed to the previously described study that enrolled only male twins, this study also included opposite-sexed DZ twins to assess differences due to sex and shared environments. A higher concordance of tinnitus was observed in MZ twins (0.32) vs. DZ twins (0.20). Concordance between DZ same-sex (0.20) and opposite-sex (0.19) twins, same-sex male DZ (0.11) vs. same-sex female DZ (0.13), as well as male MZ (0.25) vs. female MZ (0.23) were all similar. When comparing bilateral tinnitus concordance in both twin groups, a higher concordance rate was detected in MZ (0.49) vs. DZ (0.30) twins that was observed in both sexes. Younger MZ females (0.39) had a greater concordance than DZ females (0.20), but this was not observed in males. Heritability was greater in men (0.68) than in women (0.41), except when considering female twins younger than 40 years of age, where a heritability of 0.62 was determined, although it was noted by the authors that this was a highly variable group. Overall, this study concluded that while tinnitus may be environmentally driven, bilateral tinnitus may have a genetic etiology (Maas et al., 2017).

## Approaches for future genetic studies

Recently published data have begun to dissect a complex genetic basis for tinnitus. However, the general lack of consistent results is not surprising considering that the type of studies presently conducted have not been optimized through streamlined clinical patient classification criteria or by employing enhanced testing strategies of sufficient statistical power for tinnitus studies. There are many lessons that can be learned from previous analyses that include stratified patient selection and careful design of human genetic studies.

### Addressing phenotypic heterogeneity in tinnitus genetics studies

Identifying the most homogeneous tinnitus patients in terms of etiology, age, sex, severity, onset, and audiometric profile will increase study robustness by limiting genetic variance that would be expected if tinnitus is associated with these factors (Lopez-Escamez et al., 2016). Furthermore, consideration for co-occurring psychiatric disorders and quality of life evaluations are important to recognize as potentially contributing determinants (Langguth et al., 2013). The present collection of case-control association studies has grouped patients into unspecific “chronic tinnitus” cohorts from questionnaire data and only occasionally accounted for concomitant hearing loss, impeding the selection of stratified patient groups. The clinically heterogeneous nature of tinnitus makes assigning patients to one of many clinical sub-categories difficult. Current tinnitus assessments are comprised of self-report questionnaires and psychoacoustic measures (Langguth et al., 2013). There have been several classification systems proposed that consider the origin (i.e., auditory system or head and neck) or classify tinnitus according to auditory, somatosensory or psychopathology alterations (Levine and Oron, 2015). Homogeneous patient selection relies on accurate patient sub-typing; however, the present definition of tinnitus sub-types lacks consensus and is complicated by the many recognized etiologies and risk factors (Lopez-Escamez et al., 2016). The application of universal assessment protocols by clinicians would potentially benefit genetics studies in that once genetic datasets are obtained, the merging of multiple datasets by collaborating working groups can be streamlined to enhance recruitment of the several thousand patients and controls required for adequate statistical power. Furthermore, there are over 100 instruments available for clinical trial primary outcome measures, which further asserts there is a lack of tinnitus assessment consensus (Hall et al., 2016; Müller et al., 2016). Determining the standardized instruments used by practitioners would consolidate some of the assessment practices for accurate phenotyping.

### Genetic investigations in tinnitus

The unraveling of the human genome has delivered basic knowledge of a reference genome, advanced fundamental knowledge of disease architecture, and catalyzed technological advancements that fuel complex trait research. Recent developments in genetics technology and increased affordability and availability of genotyping array and sequencing data will undoubtedly propel research in the field forward. The present body of research has begun to disclose a genetic architecture for tinnitus but there is still much work ahead for the identification of specific variants influencing critical gene expression and gene products in tinnitus pathophysiology. As previously discussed, study design and patient inclusion are particularly important for a clinically heterogeneous phenotype such as tinnitus. Equally important is the method selection to support appropriate scale and resolution of data for analysis. The next section will discuss the feasibility of case-control association testing, GWAS, and twin and familial approaches in the context of tinnitus research.

### Case-control and genome-wide association testing in tinnitus

Population based association studies have long been a popular strategy to identify polymorphisms correlated with complex traits and have thus far been the most widely employed genetic study in tinnitus genetics research (Table 2). Historically, case-control candidate gene studies have not yielded abundant success and independent replications are often not possible. The same problem has also been encountered in tinnitus, which makes a reevaluation of the current and future study designs essential.

Future tinnitus association studies should overcome several problematic design setbacks. One key aspect is to exclude controls with unassessed or unrecognized tinnitus burden and select stratified patient cohorts with sufficient statistical power. Furthermore, control individuals should ideally be matched for age, sex, population background, stress/anxiety traits, and other recognized co-morbidities. Studying already characterized tinnitus patients with homogeneous tinnitus phenotypes, families with transgenerational tinnitus aggregation, or cohorts from previously performed epidemiological health studies where individuals experiencing chronic tinnitus can be re-contacted for in-depth tinnitus scoring and auditory assessment would be beneficial (Lopez-Escamez et al., 2016). Finally, drawing upon knowledge from current GWAS for complex traits such as body weight and neuropsychiatric disorders, tens to hundreds of thousands of cases and controls are required to pinpoint significant loci (Ripke et al., 2014; Locke et al., 2015). However, such studies also underscore the power of a GWAS approach for the association of common variants and genetic loci (LD blocks) with specific diseases or traits. Learning from the scale of studies that are required for other complex traits and considering the clinical complexity of tinnitus, it is likely that future studies in tinnitus need to be increased by several orders of magnitude.

### GWAS

GWAS approaches have successfully dissected the genomic architecture of complex diseases and remain a robust approach for future tinnitus research. Looking more specifically at the collection of studies presently published, it is reasonable to assume several technological advances will transform study designs. The past decade has endowed affordable sequencing technologies that have revolutionized novel gene discovery by providing an avenue from which to effectively approach complex and Mendelian disorders (Koboldt et al., 2013). Next generation sequencing (NGS), also termed high-throughput sequencing, allows parallel amplification and sequencing of the entire protein coding region of the genome (whole exome sequencing) or the entire sequence of an individual's genome (whole genome sequencing). As the majority of significant findings from GWAS are tag SNP haplotype blocks in non-coding regions and NGS provides nucleotide-resolution of scalable coding and non-coding sequence (i.e., whole exome or whole genome sequencing), NGS can be regarded as a complementary methodology to GWAS and both methods have the potential to contribute important findings to the field. This would be especially promising if a combination of causal rare coding and common non-coding regulatory variants underlie tinnitus pathology. Examples from Alzheimer and Parkinson disease research underscore this parallel approach from the implication of both common and rare variants in these diseases that were detected from GWAS and NGS approaches, respectively (Sharma et al., 2012, 2014; Guerreiro et al., 2013).

The present collection of case-control studies for tinnitus has emphasized the need for several design considerations. There has yet to be a tinnitus case-control study with adequate statistical power. Complex disorders are more challenging to detect common susceptibility alleles and require larger sample numbers to separate signal from noise and to detect frequent alleles of modest effect (Risch and Merikangas, 1996). Furthermore, population differences can have drastically differing allelic architecture that can complicate the mapping of putative risk factors when attempting association replication in patients of differing ethnicities (Sharma et al., 2014). Although underpowered, even if significance were achieved in the initial association study analyzing *GDNF* markers in German tinnitus patients, the replication study that followed studied the same three markers in a Turkish tinnitus group (Sand et al., 2012b; Orenay-Boyacioglu et al., 2016).

The only pilot tinnitus GWAS published to date in a Belgian tinnitus cohort has highlighted the worthwhile investment of this approach in tinnitus patients (Gilles et al., 2017). In light of these results, it would be worth repeating a GWAS with a stratified patient cohort of extremely severe tinnitus patients or patients who are young and therefore have a greater chance of having tinnitus due to a genetic etiology. The patients included would need to have the same tinnitus sub-type and controls should be specifically assessed for tinnitus. Replication using a tinnitus cohort would be important to highlight the same pathways and potentially achieve genome-wide significance.

### Familial aggregation and twin studies

Early studies utilizing questionnaire data from families with tinnitus returned low heritability estimates. Although both of the twin studies that have been performed each had their limitations and utilized different inclusion criteria, these recently published studies served as groundbreaking evidence suggesting tinnitus is multifactorial with both genetic and non-genetic factors contributing to its etiology (Bogo et al., 2016; Maas et al., 2017). Another central nervous system disorder, Parkinson disease, has witnessed a similar period of debate about genetic factors contributing to the disease. Early evidence disclosed low heritability estimates from twin studies (Wirdefeldt et al., 2004) and lack of familial aggregation (Levy et al., 2004), while concurrent evidence also uncovered familial aggregation (Maher et al., 2002; Payami et al., 2002; Marder et al., 2003) and heritability estimates were later calculated at 0.34 (Wirdefeldt et al., 2011). Continued research has indeed uncovered the genetic complexity of Parkinson disease. It remains to be seen whether continued research into the genetic underpinnings of tinnitus will uncover similar observations. With respect to twin studies, future studies would have to take into account the same clinical phenotyping and study design details that other genetic studies must also address.

## Future outlook

Understanding the genetic basis for tinnitus is of great public health significance that is clearly in its infancy. Many of the attempts dissecting the genetic contribution have likely been clouded by tinnitus heterogeneity that emphasizes the need for a consensus for tinnitus measures and increased sample size by several orders of magnitude. Recent heritability estimates from twin studies assert genetic factors are important in tinnitus etiology, which allows a potential understanding of basic molecular mechanisms of tinnitus and eventual diagnostic and therapeutic options. An improvement in study design will further clarify results emerging from genetics studies.

Although gene identification in tinnitus will itself represent a major advancement to the field, it will trigger several new lines of research. (1) Knowledge of candidate genes will permit basic research into the specific pathophysiological mechanisms involving animal models. A recent example is shown in a mouse knockout model with a glutamate aspartate transporter (GLAST) that leaves mice genetically susceptible to tinnitus-inducing agents such as salicylate. Such studies could contribute an understanding to how tinnitus is triggered and maintained (Yu et al., 2016). (2) Streamline effective research into drug development that profits from knowledge of specific mechanisms. (3) Clinical research into genotype-phenotype correlations can be based on knowledge of alleles involved. Furthermore, this may also enhance not only diagnostic development to support informed healthcare decisions, but also the identification of high-risk individuals to direct preventative care.

The impact from research breakthroughs analyzing the genetics of tinnitus would be enormous for tinnitus sufferers and would allow personalized and optimized therapies to be possible for tinnitus patients. The most promising data are yet to emerge and will provide much needed insights into the role of genetics in primary chronic tinnitus.

## Author contributions

BV, IN, WS, and TH participated in manuscript preparation and editing. All authors have read and approved the final manuscript.

### Conflict of interest statement

The authors declare that the research was conducted in the absence of any commercial or financial relationships that could be construed as a potential conflict of interest.
